# A meta-analysis of the effects of non-traditional teaching methods on the critical thinking abilities of nursing students

**DOI:** 10.1186/s12909-016-0761-7

**Published:** 2016-09-15

**Authors:** JuHee Lee, Yoonju Lee, SaeLom Gong, Juyeon Bae, Moonki Choi

**Affiliations:** 1 College of Nursing, Mo-Im Kim Nursing Research Institute, Yonsei University, Seoul, Korea; 2 College of Nursing, Pusan National University, Gyeongsangnam-do, Korea; 3 Yonsei University Severance Hospital, Seoul, Korea; 4 College of Nursing, Yonsei University, Seoul, Korea; 5 Department of Nursing, Bucheon University, Gyonggi-do, Korea

**Keywords:** Critical thinking, Education, Meta-analysis

## Abstract

**Background:**

Scientific framework is important in designing curricula and evaluating students in the field of education and clinical practice. The purpose of this study was to examine the effectiveness of non-traditional educational methods on critical thinking skills.

**Methods:**

A systematic review approach was applied. Studies published in peer-reviewed journals from January 2001 to December 2014 were searched using electronic databases and major education journals. A meta-analysis was performed using Review Manager 5.2. Reviewing the included studies, the California Critical Thinking Dispositions Inventory (CCTDI) and California Critical Thinking Skills Test (CCTST) were used to assess the effectiveness of critical thinking in the meta-analysis.

**Results:**

The eight CCTDI datasets showed that non- traditional teaching methods (i.e., no lectures) were more effective compared to control groups (standardized mean difference [SMD]: 0.42, 95 % confidence interval [CI]: 0.26–0.57, *p* < .00001). And six CCTST datasets showed the teaching and learning methods in these studies were also had significantly more effects when compared to the control groups (SMD: 0.29, 95 % CI: 0.10–0.48, *p* = 0.003).

**Conclusions:**

This research showed that new teaching and learning methods designed to improve critical thinking were generally effective at enhancing critical thinking dispositions.

**Electronic supplementary material:**

The online version of this article (doi:10.1186/s12909-016-0761-7) contains supplementary material, which is available to authorized users.

## Background

The medical delivery system is changing rapidly due to developments in health technology. Aging populations, complicated changes in diseases, and increases in the number of patients with advanced diseases result in diverse and high-level health needs. To satisfy these needs in the context of such changes, healthcare providers must possess skills such as critical thinking, independence, and creativity so that they can identify solutions to problems based on quick and accurate analyses [1–3]. The Institute of Medicine [4] specified Evidence-Based Practice (EBP) as a core competence for all professional healthcare providers, which by 2020 aims to apply evidence that is accurate, timely, and supported by the latest clinical research to 90 % of all clinical decisions. In the EBP process, healthcare providers are not just simple agents, but thinkers with expertise who search for and evaluate evidence to solve problems that emerge in clinical practice, subsequently making decisions to provide optimum treatment and intervention. In this process, critical thinking is vital.

Critical thinking is intentional and self-regulatory judgment that leads to interpretation, analysis, evaluation, and inference. In parallel, it produces explanations concerning whether evidence for a specific judgment is appropriate, and whether it properly considers evidential, conceptual, methodological, referential, and contextual aspects. A non-linear and cyclical process enables individuals to make decisions about what to believe and do in a given context [5]. Based on the above, Facione et al. [6] argued that to improve critical thinking, people must value the cognitive skills required for critical thinking and have the disposition to use them.

In accordance with these aforementioned changes, college education is also taking steps toward designing curricula that promote teaching and learning methods, as well as learning experiences, that use the latest technologies and information to nurture critical thinking amongst students [7]. Since the 1990s, an increasing number of colleges have begun forming curricula based on problem-based learning (PBL) and self-directed learning (SDL); likewise, since the late 2000s, the popularity of simulations and concept mapping has increased. However, a consensus has not been reached concerning the most effective teaching method for improving critical thinking. Therefore, this study aims to establish a scientific framework that will be useful for designing curricula and evaluating students in the field of nursing education and clinical practice. It seeks to achieve this by systematically examining the effects of teaching and learning methods used to improve critical thinking skills.

## Methods

This study is a meta-analysis conducted according to the systematic review guidelines established by the Cochrane Collaboration [8]. A completed PRISMA checklist is included in Additional file 1. It utilizes a quantitative approach to analyze the effect and impact of teaching and learning methods used to improve nurses’ critical thinking abilities.

This study was a meta-analysis, therefore ethics committee approval was not applicable.

### Search strategy

Studies were limited to those published from January 2001 to December 2014 in English and Korean peer-reviewed journals using the PubMed, Cochrane Library, CINAHL, Embase, and KoreaMed databases. Reference lists and major Korean academic journals were hand-searched, including the Journal of the Korean Academic Society of Nursing Education, Korean Journal of Medical Education, and the Journal of the Korean Academy of Nursing. The key search terms used included “critical thinking,” “medical,” “nursing,” “dentist,” “pharmacist,” “students,” “healthcare personnel,” “education,” and “program,” with single search terms or in combination with Boolean and wildcard.

### Inclusion criteria

First, this review considered research papers documenting randomized controlled trials or control group pre-post designs targeting healthcare providers such as doctors, dentists, nurses, and students.

Second, we selected research that used non-traditional teaching and learning methods (i.e., no lectures) for intervention. Third, we selected studies assessing critical thinking as the outcome. Finally, we selected studies using identical measurement (e.g., California Critical Thinking Dispositions Inventory [CCTDI]) and included means and standard deviations to verify effectiveness in the meta-analysis. We excluded studies in languages other than English and Korean. In addition, grey literature, such as papers that were not peer-reviewed (e.g., academic reports, dissertations), was also excluded.

### Outcome measurement

Reviewing the outcome measurements in these inclusion criteria studies, the CCTDI and California Critical Thinking Skills Test (CCTST) were used. The CCTDI consists of 75 items and 6-point Likert scale. This tool is classified into seven subscales of truth-seeking, open-mindedness, analyticity, systematicity, critical thinking confidence, inquisitiveness and maturity [6]. The target score was 350, while the cutoff score was 280 for overall disposition for CCTDI. In subscale analysis, each subscale score 30 or less represented weakness; 40, average; and 50 or above, strength [9].

The CCTST is a 34-item, multiple choice tests. This tool is classified into 5 subscales of analysis, evaluation, inference, deduction and induction [10]. The range of score in this study is 0–34, higher scores indicating higher critical thinking ability [11, 12].

### Statistical analysis

Meta-analysis was conducted using RevMan version 5.2 after related content was extracted (e.g., regarding the researcher, publication year, research design, subjects, control/experimental group teaching/learning methods, education content, education hours, measurements, and outcomes—including means and standard deviations). As studies included in the meta-analysis used some partial modification (e.g., subscale) of the CCTDI and CCTST, the standardized mean difference (SMD) using means and standard deviations was used to measure the effect size. Five studies measured the CCTDI. However, while Tiwari et al. [13] conducted three posttests, Kaveevivitchai et al. [14] conducted two; consequently, each test was analyzed separately. Thus, eight datasets were analyzed with the CCTDI.

Five studies measured the CCTST. Among these studies, Kaveevivitchai et al. [14] conducted two posttests, which were analyzed separately. Six datasets were analyzed with the CCTST.

Heterogeneity was examined by calculating using I^2^ statistics. Heterogeneity is assumed for I^2^ values of 0–73 % [15]; in such cases, a random effects model was used. The statistical meaning of the effect size was determined according to a test of overall effect and 95 % confidence intervals (CIs), based on a 5 % significance level. To verify publication bias, symmetry was examined using a funnel plot; publication bias was absent if an even distribution existed within the triangular shape.

### Quality assessment

For the final selection of literature and quality assessment, two independent researchers conducted an evaluation by applying risk of bias from Cochrane Library [8]. These seven items included the selection bias, i.e., random sequence generation and allocation concealment, performance bias, detection bias, attrition bias, reporting bias and other sources of bias. The evaluation was conducted by classifying each item as high risk of bias, low risk of bias and unclear risk of bias. In cases where researchers disagreed, decisions were eventually made based on mutual consent.

## Results

### The search findings

Following the primary search, 2534 studies were found by reviewing and hand searching the databases and references; 2309 studies remained after redundant literature was eliminated. Upon reviewing various titles and abstracts, 19 studies that satisfied each of the selection standards were identified. Of the nine studies [9, 11–14, 16–19] selected for systemic review, eight [9, 11, 13, 14, 16–19] were conducted within the realm of nursing education, while the remaining study [12] involved occupational therapy students.

**Fig. 1:**
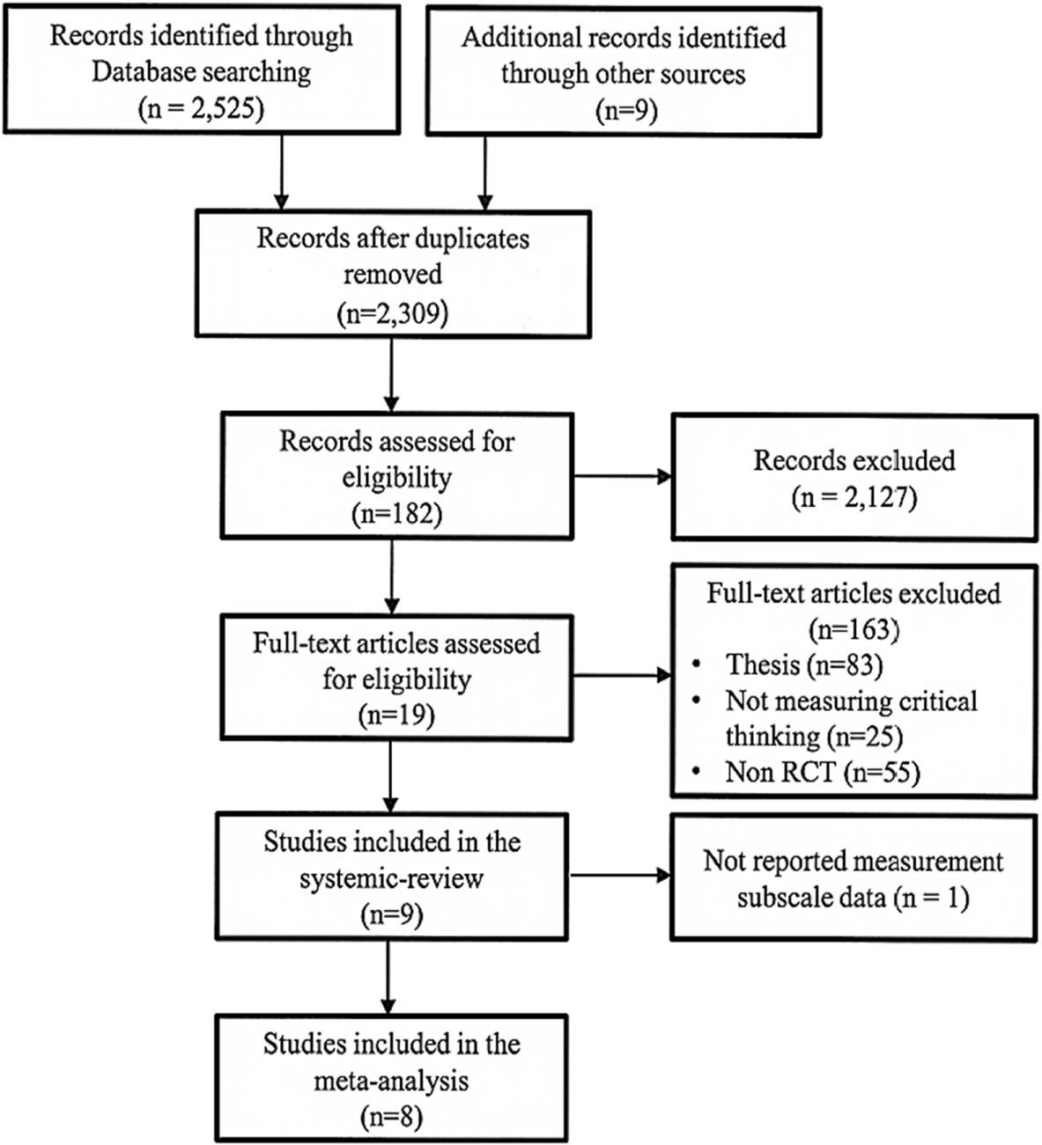
Flow chart for selection of included studies

However, Velde and colleagues [12] didn’t report measurement tool’s subscale data. Therefore, this study was excluded in this meta-analysis. Consequently, eight studies were selected for the final review (Fig. 1).

### Study quality

**Fig. 2:**
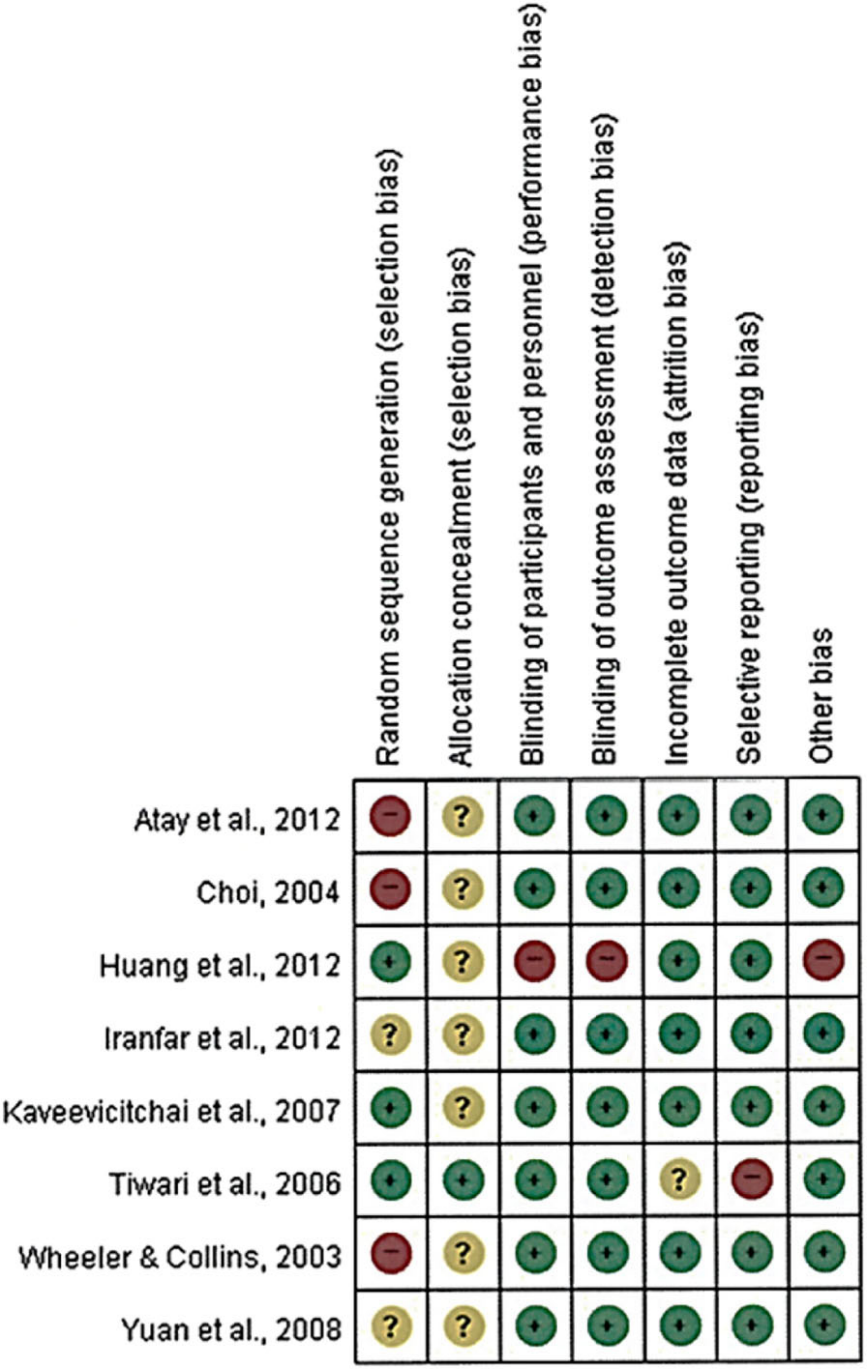
Risk of bias assessment

Overall, eight selected studies were assessed on risk of bias (Fig. 2). The results of the quality assessment revealed one study [14] satisfied six items of risk bias, six studies [11, 13, 16–19] satisfied five items, and one study [9] satisfied only three items. Three studies [11, 16, 18] were judged as having high risk of random sequence generation because these studies didn’t randomly assigned control and experimental group. Furthermore, only one study [13] had low risk on allocation concealment, while remaining seven studies didn’t reported the allocation sequence. Only one study [9] didn’t blind the intervention program to experimental group and investigator. Also during the program, participants were realized that they were observed by the researcher. Therefore, this study had a potential risk of Hawthorne effect that can produce an invalid result attributed to participants’ expectation. One study [13] might have attrition bias and reporting bias. Because the study reported selectively, i.e., mentioning effective experimental results only. Additionally, this study didn’t report missing data, which is an attrition bias.

### Study characteristics

Of the selected eight studies, one was published in 2006; four of them were published in each of 2003, 2004, 2007, and 2008; and three were published in 2012. The studies were conducted in a wide variety of countries including Korea [11], China [19], Thailand [14], Hong Kong [13], Taiwan [9], Turkey [16], Iran [17], and the United States [18].

Regarding the research design employed by the studies, four (50 %) used a randomized pretest-posttest control group design [9, 13, 14, 16], while four (50 %) used a quasi-experimental, nonequivalent pretest-posttest control group [11, 17–19]. Concerning the measurement used to measure critical thinking, three studies [13, 16, 17] used the CCTDI, three [11, 18, 19] were based upon the CCTST and two studies [9, 14] utilized both the CCTDI and CCTST.

The research subjects in most studies (6 studies; 75 %) included nursing students (midwifery students in one study), while staff nurses and nurse practitioner students were the participants in the remaining two. The range of the sample size was between 23 and 67, while the pooled sample size was 647 (experimental group = 327, control group = 320) and 452 (experimental group = 230, control group = 222) in studies that measured CCTDI and CCTST, respectively.

### Characteristics of educational method

For the teaching and learning methods used to improve the subjects’ critical thinking skills, three used PBL [11, 13, 19], three used concept mapping [9, 16, 18], one used bioscientific multimedia [14], and one used a collaborative method [17].

The intervention period varied from 8 weeks to two semesters. Regarding the PBL, Yuan et al.’s [19] implementation lasted one semester, i.e., 2 h weekly for 18 weeks, totaling 36 h. Tiwari et al.’s [13] spanned two semesters, which took 3–6 h weekly for 28 weeks. On the other hand, lessons using concept mapping were conducted for 40 min on a biweekly basis for 16 weeks [9]; alternatively, as in Wheeler and Collins’ [18] implementation, participants prepared concept maps for practical training each week during a 15-week training period following a simple orientation.

To verify the long-term effects of education, Tiwari et al. [13] measured subjects three times following intervention, while Kaveevivitchai et al. [14] measured subjects two times after intervention. The remaining seven studies measured subjects only once immediately after intervention. The characteristics of the included studies are summarized in Additional file 2.

### Results of the meta-analysis

**Fig. 3:**
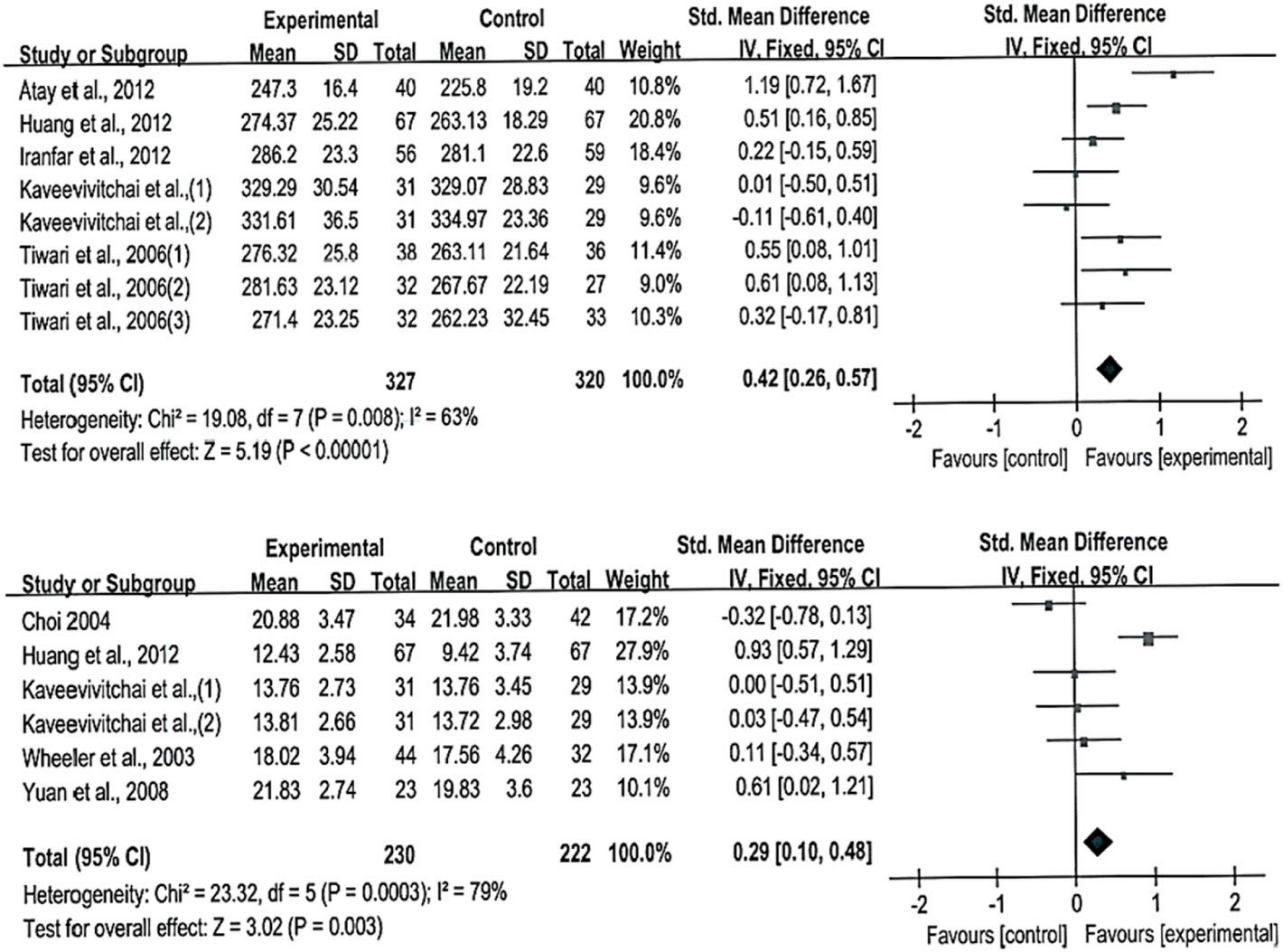
Meta-analysis and forest plot of overall CCTDI (up) and CCTST (down). CCTDI = California Critical Thinking Dispositions Inventory; CCTST = California Critical Thinking Skills Test

**Fig. 4:**
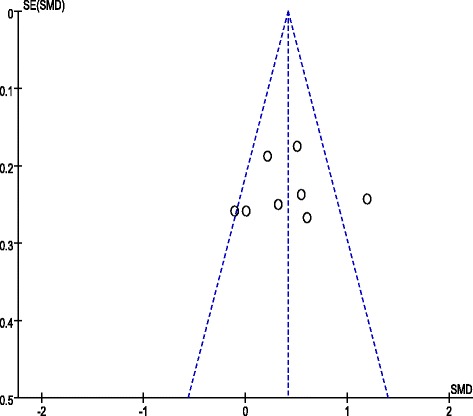
Funnel plot of CCTDI overall scores

The following are the results of the meta-analysis on the overall and subscale scores using eight and six CCTDI and CCTST outcome datasets, respectively, from each study. The eight CCTDI datasets showed moderate differences (χ^2^ = 19.08, *p* = .008, I^2^ = 63 %). The random effects model analysis revealed that the teaching and learning methods used in these studies were significantly different than the control group (SMD: 0.42, 95 % CI: 0.26–0.57, *p* < .00001; Fig. 3). The CCTDI cutoff and target scores were 280 and 350, respectively [9]. Scores of the experimental group in three studies [13, 14, 17] exhibited higher than 280 after the non-traditional educational intervention. However, each of experimental group did not reach the target score, i.e., 350. Analysis of the CCTDI subscale scores for truth-seeking (SMD: 0.32, 95 % CI: 0.01–0.47, *p* < .0001), open-mindedness (SMD: 0.37, 95 % CI: 0.22–0.53, *p <* .00001), analyticity (SMD: 0.28, 95 % CI: 0.09–0.46, *p* = .004), critical thinking confidence (SMD: 0.34, 95 % CI: 0.18–0.49, *p* < .0001), inquisitiveness (SMD: 0.36, 95 % CI: 0.21–0.52, *p* < .00001), and maturity (SMD: 0.16, 95 % CI: −0.01–0.32, *p* = 0.06) revealed a more effective increase as compared to the control group (Additional file 3). When the score of the CCTDI subscale should be higher than 50 to indicate strengthen critical thinking disposition, only one study showed a score of 50 or higher for ‘open-mindedness’ and ‘inquisitiveness’ [14]. In the funnel plot, there was symmetric shape suggesting a lack of publication bias (Fig. 4).

**Fig. 5:**
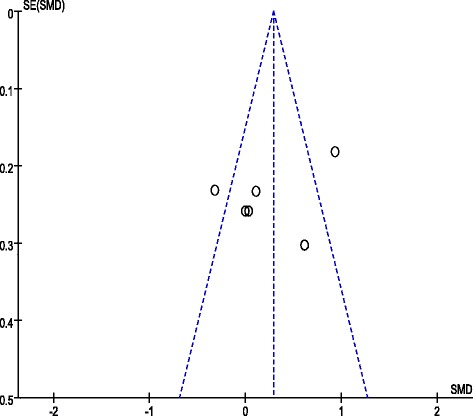
Funnel plot of CCTST overall scores

The six datasets presenting the effects of teaching and learning methods on CCTST exhibited a high level of difference (χ^2^ = 23.32, *p* = .0003, I^2^ = 79 %). Consequently, the random effects model was used for analysis, teaching and learning methods used in these studies were significant effects on the overall CCTST score when compared to the control group (SMD: 0.29, 95 % CI: 0.10–0.48, *p* = 0.003; Fig. 3). Analysis of the subscale scores, however were not revealed a more effective increase as compared to the control group (Additional file 4). Publication bias was examined using the funnel plot that revealed a symmetrical shape suggesting a lack of bias (Fig. 5).

**Fig. 6:**
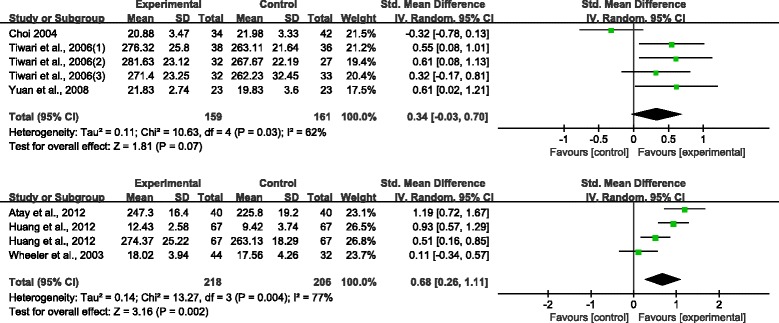
Meta-analysis and forest plot of critical thinking by educational method. *PBL (up) and Concept map (down)

Analysis of the effects of teaching and learning methods revealed that concept mapping (SMD: 0.68, 95 % CI: 0.26–1.11, *p* = 0.002, I^2^ = 77 %) was effective in improving critical thinking (Fig. 6). However, PBL (SMD: 0.34, 95 % CI: −0.03–0.70, *p* = 0.07, I^2^ = 62 %) was not significantly effective in improving critical thinking.

## Discussion

This study was conducted to verify the effects of teaching and learning methods used to improve the critical thinking of healthcare providers. As nurses must make correct judgments and efficient decisions in diverse and complex clinical situations, critical thinking is important in professional nursing. Therefore, the findings of this study are especially meaningful.

The meta-analysis revealed that diverse teaching and learning methods (i.e., concept mapping, bioscientific multimedia) are more effective than are traditional approaches in improving dispositions towards critical thinking. This result is similar with previous studies [16, 20, 21]. According to Taylor and Wros’s study [20], concept mapping was an effective visualizing learning method, especially organizing and analyzing the patient data. Concept mapping can provide the important factors as well as inter-relational knowledge, therefore, students construct the basic concept. Overall, concept mapping might be positive effect to develop students’ critical thinking.

The overall CCTST score maintained an average level of 12.4–21.8 according to Huang et al.’s [9] standard. Although critical thinking disposition significantly increased post-intervention, it did not reach a level of excellence. This indicates that it is difficult to anticipate sufficient improvement, as the intervention was performed between the first and second semester, which is a short period of time for enhancing critical thinking. Moreover, Tiwari et al. [13] measured the results three times: immediately after intervention, 1 year after, and 2 years after. The results revealed that the critical thinking disposition score gradually decreased as time passed, showing no significant difference after 2 years. This implies that sufficient effects cannot be anticipated after merely conducting education for one or two semesters in a single course, and that continuous education is needed in a variety of courses.

However, the CCTDI and CCTST are commercialized measurements that have been used in various studies. Simpson and Courtney’s research [22] asserted that these measurement tools had limitation for nursing students or nurses. Critical thinking in nursing education is the ability to assess, analyze and understand the patients’ contextual clinical situations [23]. However, these commercialized tools measured the limited aspect of critical thinking, such as analyzing and interpreting the suggested patient written data [24, 25]. These tools are not able to measure the students’ performance for example, patient specific situation driven critical thinking. Thus, these measurements were insufficient measuring the critical thinking abilities of nursing students or nurses. Furthermore, these measurements use self-report method, which may cause participants to respond in a manner that they believe society anticipates. Thus, it is necessary to interpret this study’s results with caution.

More than 20 years have passed since concepts concerning critical thinking and the education of healthcare providers have been reformed to meet the demands of outcome-based education. As the concept of critical thinking in nursing education is in constant discussion, it is necessary to consider the clinical context and the patient’s situation, not merely evaluate critical thinking skills and dispositions. Therefore, an objective measurement of critical thinking with a focus on empirically measuring student performance must be developed to determine how critical thinking should be applied in evidence-based nursing practice, and whether patients’ health problems are solved as a result.

Verifying the effectiveness of teaching and learning method showed that concept mapping was effective in improving critical thinking. This result is consistent with the previous systemic review findings in nurse education [26]. According to this study, reflective writing, concept mapping and case studies are interventions that enhance critical thinking in the context of nursing education.

In this meta-analysis, PBL was not effective in improving nursing students’ critical thinking. This finding is inconsistent with previous reports [19, 26, 27]. Recent a systemic review [28] study explained that learners’ readiness, fluency or trait, educators’ belief or attitude in critical thinking, or learning environment can bring different educational effect culturally. Kong and colleagues [27] also described that different educational method or environment can influence PBL educational effects. Additionally, Kong et al.’s [27] meta-analysis selected more than one instrument in order to examine the CCTDI and CCTST (i.e., Watson-Glaser Critical Thinking Appraisal [WGCTA], Assessment Technologies Institute Critical Thinking Test [ATI], etc.), therefore, there can be different results. However, there were insufficient evidences to support this study’s result. Thus, further studies should be conducted to examine the effectiveness of PBL on critical thinking ability.

A moderate and high degree of heterogeneity was presented in this study. We included diverse educational method to examine the effectiveness of non-traditional teaching methods on the critical thinking in this meta-analysis. Aforementioned difference can significantly affect the heterogeneity.

The quality appraisals of the eight studies equally demonstrated that an insufficient amount of research applied concealment, double blinding, and multiple study sites. The reason might be that researchers primarily functioned as educators providing the intervention. The studies did not indicate whether certain actions were taken to reduce any potential bias that may have arisen, given the issues mentioned above. Thus, to establish a solid foundation for the validity and generalization of the results, randomized controlled trials must be conducted at multiple sites by applying strict research designs.

Compared to previous studies, this study had the advantage of securing generally high-quality research for meta-analysis; this is evident in its use of studies that applied randomized controlled trials and pretest-posttest control group designs in their verification of teaching and learning methods designed to improve critical thinking.

### Limitations

There were several limitations in this study. First, only eight studies were included for meta-analysis. While visual inspection of the funnel plots revealed a symmetrical shape suggesting a lack of publication bias, the limitations of funnel plots to detect publication bias are well known, especially when the number of studies included is less than 10 and a large degree of heterogeneity exists among studies [29, 30]. Secondly, the specific intervention methods, duration, contents of the teaching and learning methods, and study quality were varied considerably by moderate to high heterogeneity reported. Thirdly, all eight studies were retrieved from the nursing literature which limits the ability of our results to be generalized to other healthcare providers.

## Conclusions

This research showed that new teaching and learning methods designed to improve critical thinking were generally effective in enhancing critical thinking dispositions. In particular, concept mapping was effective in increasing both critical thinking skills and dispositions. However, teaching and learning methods for the improvement of critical thinking must be implemented continuously throughout a curriculum. As critical thinking is an essential concept for integrated problem solving in clinical situations, it is necessary to focus on measuring capabilities in practice rather than by evaluating critical thinking by dividing it into cognitive and affective domains. Furthermore, greater effort is needed to improve research quality in order to generalize the results.

## Additional files

Additional file 1: PRISMA checklist. (DOC 64 kb) Additional file 2: Characteristics of studies included in the meta-analysis. (DOCX 25 kb) Additional file 3: Outcomes of CCTDI subscales. (DOCX 22 kb) Additional file 4: Outcomes of CCTST subscales. (DOCX 21 kb)

## Abbreviations

ATI: Assessment technologies institute critical thinking test
CCTDI: California critical thinking dispositions inventory
CCTST: California critical thinking skills test
CIs: Confidence intervals
EBP: Evidence-based practice
GRPQ: Guided reciprocal peer questioning
PBL: Problem-based learning
SDL: Self-directed learning
SMD: Standardized mean difference
WGCTA: Watson-glaser critical thinking appraisal
