# Supplementary material for: A meta-analysis of the effects of non-traditional teaching methods on the critical thinking abilities of nursing students
Source: BMC Med Educ. 2016 Sep 15;16:240. doi: 10.1186/s12909-016-0761-7 (PMC5025580; doi:10.1186/s12909-016-0761-7)
Supplement: Additional file 2: — Characteristics of studies included in the meta-analysis. (DOCX 25 kb) [file 12909_2016_761_MOESM2_ESM.docx]

**Additional file 2 Characteristics of studies included in the meta-analysis**

| **Author** | **Study Design** | **Participants** | **Intervention** | **Measurement scale** | **Results** |
| --- | --- | --- | --- | --- | --- |
| Atay et al. (2012) | RCT | Nursing students | - Experimental : Care plans using concept mapping. Total three sessions (during 3~4 hours per session). - Control : Case plans using the column format | CCTDI | Significant differences between the groups overall and subscales of CCTDI. |
| Choi (2004) | Quasi-experimental design | Nursing students | - Experimental : Five scenarios were used for PBL sessions. The experimental group conducted PBL during a school term (12 weeks). - Control : Traditional lecture | CCTST  ( Form 2000) | No significant difference between control and experimental groups on CCTST. |
| Huang et al. (2012) | RCT | Staff nurses | - Experimental :Case studies combined with Concept Maps during 16 weeks. Six case studies were used from de Castill (1999). - Control : Case studies only with same period and time | CCTST  (Form A) & CCTDI | Significant differences between the groups overall and subscales of CCTST.  Significant differences between the groups overall of CCTDI. |
| Iranfar et al. (2012) | Quasi-experimental design | Nursing and midwifery students | - Experimental : Collaborate learning(50 minutes lecture, 30 minutes group consensus, 15 minutes discussion) - Control : Individual learning | CCTDI | No significant difference between control and experimental groups in CCTDI and subscales except of inquisitiveness. |
| Kaveevivitchai et al. (2007) | RCT | Nurse practitioner students | All participants attended classes using the traditional instructional method.   - Experimental : Added 3 times of ‘Bioscientific multimedia based on 5Es inquiry cycle. - Control : Study from other resources depending on their need except multimedia. | CCTST & CCTDI | No significant difference was found between control and experimental groups on CCTDI and CCTST. Significant difference in CCTDI was found across time. |
| Tiwari et al. (2006) | RCT | Nursing students | - Experimental : 3~6 hours of PBL tutorials each week. Total 28 weeks over 2 semesters. - Control : Lecture(3~6 hours of class contact each week total of 28 weeks over 2 semesters) | CCTDI | PBL students showed significantly effectiveness in overall CCTDI. |
| Wheeler & Collins (2003) | Quasi-experimental design | Nursing Students | - Experimental : Concept mapping in accordance with specified guidelines as part of clinical preparation for every week (7.5 weeks). - Control : Traditional nursing care plans | CCTST  Pre : Form A  Post : Form B | Concept mapping is effective in helping students develop critical thinking skills. |
| Yuan et al. (2008) | Quasi-experimental design | Nursing students | - Experimental : PBL with five learning packages were held 2hours per week for 18 weeks. Control : lecture with same time and duration | CCTST  (Form A) | The PBL students had a significantly effectiveness on the overall CCTST-A, analysis, and induction subscale scores compared with the lecture students. |

CCTDI = California Critical Thinking Dispositions Inventory; CCTST = California Critical Thinking Skills Test; PBL = problem-based learning; RCT = randomized control trial
