# Supplementary material for: A meta-analysis of the effects of non-traditional teaching methods on the critical thinking abilities of nursing students
Source: BMC Med Educ. 2016 Sep 15;16:240. doi: 10.1186/s12909-016-0761-7 (PMC5025580; doi:10.1186/s12909-016-0761-7)
Supplement: Additional file 3: — Outcomes of CCTDI subscales. (DOCX 22 kb) [file 12909_2016_761_MOESM3_ESM.docx]

**Additional file 3 Outcomes of CCTDI subscales**

| **Outcomes** | **Trails** | **Sample size**  **(EG/CG)** | **Measure of**  **effects** | **Intervention**  **Effect size(CI)** | **P-value**  **of effect** | **Heterogeneity** | | | | | |  |
| --- | --- | --- | --- | --- | --- | --- | --- | --- | --- | --- | --- | --- |
|  |  |  |  |  |  | **χ^2^** | **df** | | **P-value** | | **I^2^ (%)** | |
| Truth-seeking | Atay et al. (2012), Huang et al. (2012), Iranfar et al. (2012), Kaveevivitchai et al. (2007) ^1^, Kaveevivitchai et al. (2007) ^2^, Tiwari et al. (2006) ^1^, Tiwari et al. (2006) ^2^, Tiwari et al. (2006) ^3^ | 647  (327/320) | SMD | 0.32  (0.16, 0.47) | < 0.0001 | 25.23 | 7 | 0.0007 | | 72 | |  |
| Open-mindness | Atay et al. (2012), Huang et al. (2012), Iranfar et al. (2012), Kaveevivitchai et al. (2007) ^1^, Kaveevivitchai et al. (2007) ^2^, Tiwari et al. (2006) ^1^, Tiwari et al. (2006) ^2^, Tiwari et al. (2006) ^3^ | 647  (327/320) | SMD | 0.37  (0.22, 0.53) | < 0.00001 | 9.44 | 7 | 0.22 | | 26 | |  |
| Analyticity | Atay et al. (2012), Huang et al. (2012), Iranfar et al. (2012), Kaveevivitchai et al. (2007) ^1^, Kaveevivitchai et al. (2007) ^2^ | 449  (225/224) | SMD | 0.28  (0.09, 0.46) | 0.004 | 8.46. | 4 | 0.08 | | 53 | |  |
| Systematicity | Atay et al. (2012), Huang et al. (2012), Iranfar et al. (2012), Kaveevivitchai et al. (2007) ^1^, Kaveevivitchai et al. (2007) ^2^ | 449  (225/224) | SMD | 0.12  (-0.07, 0.30) | 0.31 | 4.78 | 4 | 0.31 | | 16 | |  |
| Critical  thinking  confidence | Atay et al. (2012), Huang et al. (2012), Iranfar et al. (2012), Kaveevivitchai et al. (2007) ^1^, Kaveevivitchai et al. (2007) ^2^, Tiwari et al. (2006) ^1^, Tiwari et al. (2006) ^2^, Tiwari et al. (2006) ^3^ | 647  (327/320) | SMD | 0.34  (0.18, 0.49) | < 0.0001 | 14.44 | 7 | 0.04 | | 52 | |  |
| Inquisitiveness | Atay et al. (2012), Huang et al. (2012), Kaveevivitchai et al. (2007) ^1^, Iranfar et al. (2012), Kaveevivitchai et al. (2007) ^2^, Tiwari et al. (2006) ^1^, Tiwari et al. (2006) ^2^, Tiwari et al. (2006) ^3^ | 647  (327/320) | SMD | 0.36  (0.21, 0.52) | < 0.00001 | 8.24 | 7 | 0.31 | | 15 | |  |
| Maturity | Huang et al. (2012), Iranfar et al. (2012), Kaveevivitchai et al. (2007) ^1^, Kaveevivitchai et al. (2007) ^2^, Tiwari et al. (2006) ^1^, Tiwari et al. (2006) ^2^, Tiwari et al. (2006) ^3^ | 567  (287/280) | SMD | 0.16  (-0.01, 0.32) | 0.06 | 8.62 | 6 | 0.20 | | 30 | |  |

EG= Experimental group, CG= Control group, ^number^ frequency of post test
