# Supplementary material for: A meta-analysis of the effects of non-traditional teaching methods on the critical thinking abilities of nursing students
Source: BMC Med Educ. 2016 Sep 15;16:240. doi: 10.1186/s12909-016-0761-7 (PMC5025580; doi:10.1186/s12909-016-0761-7)
Supplement: Additional file 4: — Outcomes of CCTST subscales. (DOCX 21 kb) [file 12909_2016_761_MOESM4_ESM.docx]

**Additional file 4 Outcomes of CCTST subscales**

| **Outcomes** | **Trails** | **Sample size**  **(EG/CG)** | **Measure of**  **effects** | **Intervention**  **Effect size(CI)** | **P-value**  **of effect** | **Heterogeneity** | | | |
| --- | --- | --- | --- | --- | --- | --- | --- | --- | --- |
|  |  |  |  |  |  | **χ^2^** | **df** | **P-value** | **I^2^ (%)** |
| Analysis | Choi. (2004), Huang et al. (2012), Kaveevivitchai et al. (2007) ^1^, Kaveevivitchai et al. (2007) ^2^, Wheeler et al. (2003) , Yuan et al. (2008) | 452  (230/222) | SMD | 0.27  (-0.08, 0.61) | 0.13 | 16.01 | 5 | 0.007 | 69 |
| Evaluation | Choi. (2004), Huang et al. (2012), Kaveevivitchai et al. (2007) ^1^, Kaveevivitchai et al. (2007) ^2^, Wheeler et al. (2003) , Yuan et al. (2008) | 452  (230/222) | SMD | 0.07  (-0.23, 0.37) | 0.65 | 12.47 | 5 | 0.03 | 60 |
| Inference | Choi. (2004), Huang et al. (2012), Kaveevivitchai et al. (2007) ^1^, Kaveevivitchai et al. (2007) ^2^, Wheeler et al. (2003) , Yuan et al. (2008) | 452  (230/222) | SMD | 0.14  (-0.11, 0.38) | 0.28 | 8.51. | 5 | 0.13 | 41 |
| Deduction | Choi. (2004), Huang et al. (2012), Kaveevivitchai et al. (2007) ^1^, Kaveevivitchai et al. (2007) ^2^, Wheeler et al. (2003) , Yuan et al. (2008) | 332  (168/164) | SMD | 0.28  (-0.07, 0.30) | 0.27 | 13.92 | 3 | 0.003 | 78 |
| Induction | Choi. (2004), Huang et al. (2012), Kaveevivitchai et al. (2007) ^1^, Kaveevivitchai et al. (2007) ^2^, Wheeler et al. (2003) , Yuan et al. (2008) | 332  (168/164) | SMD | 0.23  (-0.15, 0.60) | 0.23 | 8.26 | 3 | 0.04 | 64 |

EG= Experimental group, CG= Control group, ^number^ frequency of post test
